# Editorial: Microbial Biotechnology Providing Bio-based Components for the Food Industry

**DOI:** 10.3389/fmicb.2019.02843

**Published:** 2019-12-05

**Authors:** Laurent Dufossé, Mireille Fouillaud

**Affiliations:** Chemistry and Biotechnology of Natural Products, Université de la Réunion, ESIROI Agroalimentaire, Saint-Denis, France

**Keywords:** food, ingredient, fermentation, enzyme, peptide, colorant, bacteriocin, thaumatin

This Frontiers Research Topic provides an inter- and multi-disciplinary platform for reviews and researches dedicated to microbial biotechnology providing bio-based components for the food industry. The findings presented in this special issue give a foundation for enlarging the current exploitation of the metabolic diversity in fungi, yeasts, bacteria, and microalgae for improved production of food and other industrial products. Thus, this topic did appeal not just to those interested in the screening and metabolic investigation of microorganisms but also to the industrial biotechnology, the process optimization, the fermentation technology, and the bio-products research community.

Ingredients derived from microbial fermentation or extracted from microalgae are steadily gaining ground in the food industries (Dufossé, 2018). Thickening or gelling agents (e.g., polysaccharides such as xanthan, curdlan, gellan), flavor enhancers (yeast hydrolysate, monosodium glutamate), lipids (polyunsaturated fatty acids—PUFAs, sterols), flavor compounds (gamma-decalactone, diacetyl, methyl-ketones), vitamins, essential amino acids, pigments/colorants (carotenoids, azaphilones) (Dufossé et al., 2014; Venil et al., 2014), surfactants and acidulants (lactic acid, citric acid) are some examples illustrating this trend of the bio-based economy. Efforts have been made and continue to be done in order to reduce the production costs of components produced by algal ponds and microbial fermentation, since synthetic ones or those extracted from natural plant sources can often be produced more economically. Fungi, yeasts, bacteria, and microalgae are considered as promising living organisms for sustainable, large-scale production of commodities such as food, feed, chemicals, materials, and biofuels.

The special issue emphasizes the crucial role that microorganisms and microalgae are currently playing and are likely to continue to play in future as microbial cell factories for the production of food grade components and bio-based ingredients in general. This is due to the versatility in their metabolic pathways and biochemical profiles, amenability for easy large-scale cultivation, and a long history of production by well-investigated production strains. Topics broadly cover studies in Screening and selection, Molecular traits, Metabolic investigation and regulation, Analytical chemistry, Physiology and biochemistry, Process optimization, Fermentation, Extraction techniques, Biomass and bio-products, Cultivation technology, Formulation and applications.

Joseph et al. have reviewed the current state and the perspectives of bioproduction of the recombinant sweet protein thaumatin, which is one of the most promising alternatives for sugar and artificial sweeteners. Recombinant DNA technology is used in the most favorable host known today, the methylotrophic yeast, *Pichia pastoris*.

Sen et al. have provided comprehensive information about challenges and the way forward for application of microbial pigments in the food industry.

In the same pigments and colorants scientific field, Liu et al. have examined the diversity of chemical structures from *Monascus*, including chemical modification of orange *Monascus* pigments and fine analyses of commercial red and yellow *Monascus* pigments present in Chinese market.

As a global approach Kallscheuer wrote a systematic study about engineered microorganisms for the production of food additives approved by the European Union. Currently, the list of substances authorized by the European Food Safety Authority (EFSA) (referred to as “E numbers”) comprises 316 natural or artificial substances including small organic molecules, metals, salts, but also more complex compounds such as plant extracts and polymers. It is impressive that a broad range of different compounds ranging from small organic acids to more complex secondary metabolites or polymers such as oligopeptides can now be accessed by tailor-made microbial cell factories.

Many articles of this special issue emphasize the power of microorganisms which are able to modify, to improve the properties of many food products or by-products: (i) production from whey of peptides with bacterial antivirulence effects (Ali et al.), (ii) wheat, rice, corn, and amaranth flour proteins treated with microbial transglutaminase, followed by immunoreactivity testing of gluten-sensitized sera toward modified flours (Scarnato et al.), (iii) bioconversion of beet molasses to alpha-galactosidase and ethanol (Álvarez-Cao et al.), (iv) production of fructooligosaccharides from aguamiel, the sap from agave plants (Picazo et al.), or (v) degradation of toxic steroidal glycoalkaloids from potato juice, a by-product of the potato industry, of the starch processing (Hennessy et al.).

Two papers focused on beneficial effects of microorganisms and microbial metabolites on food preservation. First one on the biopreservative efficacy of *Lactobacillus rhamnosus* bacteriocin on stored fish filets (Sarika et al.) and the second one on the induction of malolactic fermentation of Patagonian Malbec wine with blend cultures of native *Lactobacillus plantarum* and *Oenococcus oeni* strains (Brizuela et al.).

Fine tuning of primary and secondary microbial metabolisms is also of crucial importance, as shown by Liu et al. with small GTPases involved in *Monascus ruber*, a filamentous fungi used for more than a thousand years in Asia for the production food ingredients or as demonstrated by Sgobba et al. who genetically modified *Corynebacterium glutamicum* for utilization of alternative feedstocks such as pentose sugars or hexosamines, to produce the amino acids L-glutamate, L-lysine, and the carotenoid lycopene.

Taken all together, all the papers gathered in this Research Topic illustrate how microbial cell factories (native, engineered, or heterologous) are able to provide at an industrial scale biobased components for the food industry.

Last but not least, we, as Frontiers Guest Editors would like to deeply thanks all the referees for their huge input in handling the manuscripts, and the Frontiers Editorial team.

## Author Contributions

All authors listed have made a substantial, direct and intellectual contribution to the work, and approved it for publication.

### Conflict of Interest

The authors declare that the research was conducted in the absence of any commercial or financial relationships that could be construed as a potential conflict of interest.
